# The New Therapeutic Frontiers in the Treatment of Eosinophilic Esophagitis: Biological Drugs

**DOI:** 10.3390/ijms25031702

**Published:** 2024-01-30

**Authors:** Erminia Ridolo, Alessandro Barone, Martina Ottoni, Silvia Peveri, Marcello Montagni, Francesca Nicoletta

**Affiliations:** 1 Department of Medicine and Surgery, University of Parma, 43121 Parma, Italy; 2 Departmental Unit of Allergology, Guglielmo da Saliceto Hospital, 29121 Piacenza, Italy

**Keywords:** biological drugs, benralizumab, cendakimab, dupilumab, eosinophilic esophagitis, lirentelimab mepolizumab, monoclonal antibodies, reslizumab, tezepelumab

## Abstract

Eosinophilic esophagitis (EoE) is a multifaceted disease characterized by a wide heterogeneity of clinical manifestations, endoscopic and histopathologic patterns, and responsiveness to therapy. From the perspective of an effective approach to the patient, the different inflammatory mechanisms involved in the pathogenesis of EoE and biologics, in particular monoclonal antibodies (mAbs), targeting these pathways are needed. Currently, the most relevant is dupilumab, which interferes with both interleukin (IL)-4 and IL-13 pathways by binding IL-4 receptor α, and is the only mAb approved by the European Medicine Agency and US Food and Drug Administration for the treatment of EoE. Other mAbs investigated include mepolizumab, reslizumab, and benralizumab (interfering with IL-5 axis), cendakimab and dectrekumab (anti-IL-13s), tezepelumab (anti-TSLP), lirentelimab (anti-SIGLEG-8), and many others. Despite the undeniable economic impact of biologic therapies, in the near future, there will be room for further reflection about the opportunity to prescribe biologic agents, not only as a last-line therapy in selected cases such as patients with comorbidities involving common pathways. Although recent findings are very encouraging, the road to permanent success in the treatment of EoE is still long, and further studies are needed to determine the long-term effects of mAbs and to discover new potential targets.

## 1. Introduction

Eosinophilic esophagitis (EoE) is a chronic antigen-driven non-IgE inflammatory disease of the esophagus characterized by a dysregulated T helper 2 (Th2) immune response resulting in epithelial remodeling and esophageal dysfunction [1]. According to a recent meta-analysis, the prevalence of EoE is 42.2/100,000 for adults and 34/100,000 for children, while the incidence is 6.6/100,000/year in children and 7.7/100,000/year in adults [2], with these numbers expected to increase due to the growing awareness of this condition. Clinical manifestations usually depend on the age at onset; children usually present nonspecific symptoms or signs such as feeding difficulties, heartburn, vomiting, abdominal pain, and failure to thrive. On the other hand, adolescents and adults are more likely to report symptoms related to esophageal fibrosis, such as dysphagia and food impactions [3,4]. The recognition of this condition may be challenging because of compensatory mechanisms typically employed by patients to ease the bolus transit, i.e., avoidance of harder foods, smaller bites, longer chewing, and drinking frequently during the meal, thus leading to an underestimation of the clinical presentation and to a disadvantageous delay in diagnosis and treatment [3,4]. Predictably, restrictions on daily life caused by EoE have a significant negative impact on the quality of life of both patients and their caregivers/families, as well as on medical costs [5].

Typical endoscopic findings in patients affected by EoE are edema (decreased vasculature of mucosa), exudates (whitish plaques), furrows (vertical lines within the mucosa), trachealization (concentric rings of esophageal narrowing), and strictures [3]. The confirmation of the diagnosis is histologic, requiring ≥15 eosinophils per high-powered field reported from a minimum of six biopsies executed on both proximal and distal esophagus [6].

The conventional treatment for EoE includes high-dose proton-pump inhibitors (PPIs), swallowed topical steroids, elemental and elimination diets, and esophageal dilation [3]. Among the swallowed topical steroids, budesonide is approved by the European Medicine Agency (EMA) [7]. Currently, the only therapy approved by both the US Food and Drug Administration (FDA) and EMA for adolescents and adults is the biologic agent dupilumab [8,9].

In light of all this, EoE is clearly a multifaceted disease characterized by a wide heterogeneity of clinical manifestations, endoscopic patterns, comorbidities, evolution, and responsiveness to therapy, all depending on the patient-specific immunopathologic condition. In the perspective of an effective approach to EoE, herein, the different inflammatory mechanisms involved in the pathogenesis are deepened, with a specific regard for biologics, in particular monoclonal antibodies (mAbs), targeting these pathways.

## 2. Pathogenic Mechanisms of Eosinophilic Esophagitis

EoE is a Th2 disease characterized by a dysregulated inflammatory cascade in response to dietary and, to a lesser extent, aero-allergens, stimulating epithelial cells to secrete alarmins (cytokines including, i.e., IL-25, IL-33, and thymic stromal lymphopoietin TSLP), which promote the generation of Th2 cells and the activation of epithelial, dendritic, and other immune cells residing in the esophageal mucosa [10]. Recurring mediators involved in this inflammatory milieu are, inter alia, IL-4, IL-5, IL-13, eotaxin, and periostin, triggering eosinophil infiltration and mast cell activation [10]. Particularly, eosinophils are the hallmark of the pathogenesis of EoE, releasing cytotoxic proteins and mediators of inflammation, such as eosinophil peroxidase, eosinophil cationic protein, and major binding protein, leading both to tissue damage and esophageal dysmotility due to muscarinic M2 receptors dysfunction [11]. Among all cytokines, IL-5 is the most important for the proliferation, survival, and activation of eosinophils. Regarding mast cells, although their concentration in the esophageal mucosa and their release of transforming growth factor β1 (TGF-β1), tryptase, leukotrienes, prostaglandins, and histamine have been proven, the trigger of their activation in EoE is still unknown, likely not to be recognized in IgE. As a result of such inflammation, esophageal mucosa is subject to an increased permeability leading to higher exposure to insulting antigens, thus resulting in a self-perpetuating process [11].

It is well-known that the esophageal epithelium and sub-epithelium undergo extensive remodeling, developing dilated intracellular spaces (DIS), basal zone hyperplasia (BZH—an expansion of the basal cell layer that can exceed 15% of the total epithelial thickness [12]), fibrosis, angiogenesis, and smooth muscle hyperplasia [12]. DIS of the esophageal epithelium, also known as spongiosis, is a morphologic feature of patients with EoE, and it is usually responsive to elimination diets and steroid therapy. Mechanisms at the basis of DIS are different and still not fully understood. Upregulation of the SLC9A3 gene encoding NHE3 (sodium–hydrogen exchanger member 3) in the suprabasal layer of the esophageal epithelium has been found to correlate with esophageal eosinophilic inflammation and DIS in samples of patients with EoE. Moreover, in vitro IL-13 stimulation of primary esophageal epithelial cells increased NHE function, and its pharmacologic inhibition through the humanized anti-IL-13 monoclonal antibody QAX576 substantially decreased the area of DIS formation [13].

The esophageal mucosa of active EoE patients contains activated Th2 cells that secrete increased levels of the inflammatory cytokine LIGHT, a member of the tumor necrosis factor (TNF) superfamily that promotes the presence of resident esophageal mucosal fibroblasts and their migration to the epithelium, where they directly interact with eosinophils. Moreover, LIGHT promotes TGF-β-driven myofibroblast differentiation, thus favoring the onset of the fibrostenotic phenotype in a self-perpetuating way [14]. Particularly, TGF-β is linked with the production of collagen, and it can induce smooth muscle proliferation, hyperplasia, and contraction, leading to esophageal dysmotility [15]. TGF-β1 is released by several inflammation cells in the esophagus [16], and although it has involvement in wound healing, its continuous expression in EoE might act as a driver of pathologic remodeling, i.e., by increasing the fibroblast expression of herpes virus entry mediator (HVEM) [14], a receptor for LIGHT involved through the nuclear factor kappa B2 (NF-κB2) signaling pathway in pro-inflammatory and anti-homeostatic gene expression [17]. In this regard, a recent in vitro study found an inhibitor of the NF-κB pathway, NIK-SMI1, is able to reduce the expression of several LIGHT-induced genes, thus speculating a possible protection of healthy fibroblasts [17]. Another in vitro study employing esophageal biopsy tissue from EoE patients put the focus on mucosal lysyl oxidase, an extracellular collagen cross-linking enzyme, describing its upregulation as potentially involved in the EoE fibrostenosis [18].

A cross-sectional study on 185 patients (3–70 years old) conducted by the Consortium of Eosinophilic Gastrointestinal Disease Researchers (CEGIR) was conducted to elaborate an endotype classification of EoE starting from molecular, clinical, and histopathologic analysis. Three main endotypes were recognized: EoE1, EoE2, and EoE3. Particularly, surface epithelial alteration and BZH were significantly higher in EoE2 and EoE3 compared to EoE1, respectively. More specifically, EoEe1 prevails in atopic, steroid-responsive patients, and it corresponds to relatively mild histologic, endoscopic, and molecular changes, with a significant association with normal-appearing esophagus and an inverse relation to a history of esophageal dilation. EoEe2, more prevalent in patients with younger onset, was characterized by the highest expression of cytokines (especially those involved in type 2 immune responses, i.e., IL-4 and TSLP) and steroid-refractoryness. EoEe3, characterizing non-atopic adult-onset patients, was associated with a fibrostenotic pattern, showing the highest degree of endoscopic and histologic severity and the lowest expression of epithelial differentiation genes [19].

## 3. Biological Drugs in EoE and Their Targets

### 3.1. Dupilumab: The Anti-IL-4/IL-13 mAb

Dupilumab is a humanized IgG4 monoclonal antibody that binds the α subunit of the heterodimeric IL-4 receptor [20]. IL-4Rα may link to the γc chain, forming IL-4R type I (binding exclusively IL-4 and being expressed only on hematopoietic cells), or it may associate the receptor for IL-13, IL-13Rα1, forming IL-4R type II (binding both IL-4 and IL-13 and being expressed on both hematopoietic and epithelial cells) [21]. The pivotal role played by IL-4 and IL-13 released by type 2 cells in target tissues of atopic diseases, such as lung, skin, and gut, is very well-known [22,23]. As regards EoE, IL-4 and IL-13 are involved in favoring the releasing of eotaxin (CCL26) by esophageal epithelial cells, leading to eosinophils recruitment [24], Th2 differentiation, B cell IgG1 and IgE class-switching, and in epithelial to mesenchymal transition with consequent fibrosis [11,25]. Being more abundant than IL-4 in EoE, IL-13 plays a key role in the pathogenesis of EoE [26]. By binding its receptors expressed on esophageal epithelial cells (IL-4-Rα, IL-13-Rα1, and IL-13-Rα2) [26], IL-13 promotes esophageal barrier dysfunction by virtue of regulating the expression of many pivotal actors such as calpain 14, desmoglein-1, and synaptopodin [27,28]. Particularly, in vitro experiments conducted on cultured primary human esophageal cells found IL-13 to be related to altered patterns of tight junctions (intercellular junctional complexes on esophageal epithelial cells) and to mediate a down-regulation of the filaggrin expression, a key protein in the bond of the cytoskeleton of contiguous cells [29]. Furthermore, IL-13 overexpression leads to epithelial–mesenchymal transition, characterized by lower expression of tight junctions and E-cadherins and higher expression of depolarized cytoskeletal proteins (both typical of the epithelial cells), and to increased collagen deposition in the esophageal extracellular matrix (more typical of activated fibroblasts and myofibroblasts) [30,31]. As suggested in in vivo experiments on an eosinophil-deficient mouse with IL-13 overexpression, IL-13 may drive esophageal fibrosis independently of its role in eosinophil recruitment [32].

By targeting and blocking such a strategic pathway, dupilumab has proved to attenuate or abrogate disease chronicity and severity so much that, currently, it is the only biologic agent approved by both the EMA and FDA for the treatment of EoE in patients over 12 years of age and 40 kg of weight. Unlike in the US, duplumab can be employed in Europe only in case of failure of or contraindications to conventional EoE treatments [8,9].

A recent phase 3 trial proved the effectiveness of weekly subcutaneous administrations of dupilumab in the improvement of both histologic and clinical outcomes in patients 12 years or older with a documented diagnosis of EoE despite at least 8 weeks of therapy with PPIs [33]. More specifically, this trial was conducted in three parts. In part A, patients underwent a 1:1 randomization (subcutaneous administration of dupilumab 300 mg weekly vs. placebo), while in part B, a 1:1:1 randomization (subcutaneous administration of dupilumab 300 mg weekly vs. subcutaneous administration of dupilumab 300 mg every two weeks vs. placebo); both parts A and B lasted 24 weeks. In part C, all patients who completed parts A or B, including the ones in the placebo groups, were administered dupilumab 300 mg subcutaneously every week up to week 54. In both parts A and B, a significant histologic complete remission (≤6 eosinophils/hpf) was shown in all groups involving patients administered with dupilumab compared to placebo. In part C, patients of parts A and B who were administered dupilumab weekly had a sustained remission even at week 54, and those who were part of the placebo group reported a significant remission [33]. As regards the Dysphagia Symptom Questionnaire (DSQ), in parts A and B, a significant improvement was reported only in the “weekly administration” groups, unlike patients in part B, administered every two weeks, thus suggesting a role of the serum concentration of dupilumab in the effectiveness of the treatment (higher in weekly interval of administration) [33]. The most reported adverse effects were injection-site reactions [33], likewise in the previous phase 2 trial, where nasopharyngitis was also reported as common [34]. In both of the trials, no trends to hypereosinophilia were reported [33,34]. Another recent trial assessed the real-world efficacy of dupilumab (weekly subcutaneous administration of 300 mg) in severe, refractory to standard therapies and fibrostenotic EoE, reporting a significant increase in the pre-dilation esophageal diameter (from 13.9 to 16.0 mm) and global symptoms improvement in 91% of patients (respectively, *p* < 0.001) [35]. All of these major studies on dupilumab involved patients affected by EoE refractory to traditional therapies; nevertheless, the failure of previous therapies for its prescription is not required by the FDA, and this could be very interesting from the perspective of prescribing this biologic agent as first-line therapy in selected cases, i.e., for patients with low adherence to dietary elimination or patients characterized by multiple co-morbid atopic conditions (such as asthma, atopic dermatitis, or chronic rhinosinusitis with nasal polyps—CRSwNP) for which dupilumab is also approved [36].

Further details about the potential employment of dupilumab will come from ongoing studies of pediatric patients (1–11 years; 5–60 kg) (ClinicalTrials.gov Identifier: NCT04394351) [37], of long-term (124 weeks) esophageal function and remodeling (ClinicalTrials.gov Identifier: NCT06101095) [38], and of facilitating the re-introduction of foods that trigger EoE such as milk, egg, wheat, and soy (ClinicalTrials.gov Identifier: NCT05247866) [39].

### 3.2. Cendakimab and Dectrekumab: The Anti-IL-13 mAbs

Cendakimab, also known as RPC4046 and CC-93538, is an IgG1k humanized monoclonal antibody that recognizes IL-13 and inhibits its binding to both IL-13-receptor specific subtypes (IL-13-Rα1 and IL-13-Rα2) [40]. A multicenter study (HEROES) was performed on adult patients with active EoE treated with weekly subcutaneous administrations of 180 mg, 360 mg of cendakimab, or placebo. Significant improvements in both histopathologic (<6 eosinophils/hpf and Eosinophil Histologic Scoring System—EoEHSS) and endoscopic (EREFS) outcomes at week 16 were reported in patients treated with cendakimab compared to placebo. Regarding the clinical outcomes, no significant amelioration in dysphagia was achieved, but in the high-dose group, a significant positive effect on the global assessment of disease severity score was reported. The most common adverse events reported in this study were headache and upper respiratory tract infections [41], the latter confirmed, together with nasopharyngitis, as the most reported in the open-label extension (52 weeks) of the trial [42]. More specifically, all patients who completed the double-blind induction phase were administered cendakimab 360 mg weekly, achieving a positive trend in symptom remission (symptom-based EoE activity index score ≤ 20) in all three previous groups [42]. Furthermore, esophageal samples obtained from patients in the HEROES trial were characterized for their epithelial–mesenchymal transition at week 16, and a trend in the mean percentage of vimentin-positive cells that was inversely proportional to the dose administered of cendakimab was found. On the contrary, E-cadherin expression was increased, thus, as a whole, suggesting a potential role of IL-13 inhibition in a reduction in fibrostenotic risk in EoE [43].

More clarifications about such promising results will come from three more ongoing studies regarding cendakimab. One is a double-blind trial involving not only adults but also adolescents (ClinicalTrials.gov Identifier: NCT04753697) [44], for which is already planned an open label long-term extension (ClinicalTrials.gov Identifier: NCT04991935) [45], while another one investigates drug–drug interactions with selected cytochrome P450 substrates (ClinicalTrials.gov Identifier: NCT05175352) [46].

Dectrekumab, also known as QAX576, is a human investigational anti-IL-13 mAb with anti-inflammatory potential, already considered not only for other Th2 diseases but also for the treatment of Crohn’s disease and keloids [47]. The proof-of-concept trial performed to evaluate the effectiveness of dectrekumab in EoE treatment involved adult patients who were administered 3 monthly endovenous infusions of dectrekumab 6 mg/kg or placebo, with the possibility of an open-label extension for an additional 3 monthly infusions. Despite a significant reduction in mean esophageal eosinophil count in the treatment arm compared to placebo (−60% vs. +23%, respectively) sustained up to 6 months, the primary endpoint (responder rate for a greater than 75% decrease in peak eosinophil counts) was not met. Similarly, despite a trend for improved frequency and severity of dysphagia (assessed through the Mayo Dysphagia Questionnaire—MDQ), this was not statistically meaningful. Interestingly, an improved expression of mediators critically involved in the EoE-related inflammation was reported, such as eotaxin-3, periostin, and markers of mast cells and barrier dysfunction [48].

### 3.3. Mepolizumab, Reslizumab and Benralizumab: The Anti-IL-5 mAbs

IL-5 and its receptor (IL-5-R) have been interesting targets for the management of patients affected by EoE for a long time. IL-5 is the most specific eosinophilic cytokine. It is secreted by Th2 cells, mast cells, innate lymphoid cells of type 2 (ILC2s), and eosinophils, and by binding the α subunit of IL-5-R (CD125), it modulates eosinophilic activities from the proliferation of their progenitors to priming of cytotoxicity by mature cells and delaying their apoptosis [49]. As a consequence, the prolonged release of pro-fibrotic factors such as TGF-β1 and fibroblast growth factor 9 (FGF-9) is very strategic in all those processes underlying epithelial remodeling and esophageal dysmotility (i.e., basal zone hyperplasia, fibrosis of the lamina propria, and expansion of muscularis propria) [50]. A study conducted on the esophageal specimens of 312 patients highlighted a non-direct proportion between higher levels of IL-5 and both histological and endoscopic abnormalities. More specifically, higher expression of IL-5 in active cases of EoE was found, but without a linear transition. Patients passed, in fact, from an IL-5 low milder phenotype to an IL-5 high phenotype developed in response to inflammatory or antigenic triggers, up to an IL-5 intermediate phenotype more typical of the latter fibro-stenosing phase of the disease [51].

Mepolizumab is an IgG1k humanized monoclonal antibody that, as well as the IgG4k humanized mAb reslizumab, binds circulating IL-5 and, therefore, prevents its bond to IL-5R [49]. In the first trial concerning the use of an anti-IL-5 mAb in EoE, mepolizumab was administered (3 monthly infusions of 750 mg) to four adults with a history of long-standing EoE and oesophageal strictures. A significant reduction in the mean oesophageal eosinophilia (about 9-fold) has been reported, but never under the threshold level for the complete EoE remission (<5 eosinophils/hpf). As regards the improvements described in clinical outcomes (i.e., dysphagia) and quality of life scores, it is difficult to verify if they resulted from the biologic treatment or the previous and concomitant traditional therapy with steroids, PPIs, and elimination diet [52]. Latter trials performed on mepolizumab, in fact, confirmed a trend regarding histopathologic improvements, but they never [53] or rarely (5/57 patients) [54] achieved complete histopathologic remission, even despite higher doses (2 weekly infusions of 750 mg followed by 2 more infusions of 1500 mg at the 5th and 9th weeks) [53]. Furthermore, patients did not report a significant symptomatic improvement [53,54]. Lastly, a phase 2 trial investigating the effectiveness of mepolizumab via subcutaneous route confirmed the trend performed by this mAb, which is a discrepancy between the reduction in the eosinophilic infiltration of the oesophageal epithelium and a trend to non-progression of the endoscopic outcomes, compared to the lack of tangible improvements in the clinical picture assessed through the EoE Symptom Activity Index (EEsAI) [55].

Unfortunately, a study on reslizumab involving children and adolescents with EoE did not help much in denying what was already stated for mepolizumab, at least in the short term. Once again, the anti-IL-5 provided a significant reduction in intraepithelial esophageal eosinophilia without significant clinical improvements [56]. On the contrary, another small trial provided a larger vision of the long term by considering a follow-up of 9 years in which patients all showed a considerable clinical amelioration (absence of vomiting), with none of them reporting esophageal narrowing or strictures and achieving a complete histopathologic remission [57].

Benralizumab is an IgG1k mAb directed to the α subunit of IL-5-R [49], which received orphan drug designation status from the FDA for the treatment of EoE. Compared to the anti-IL-5 biologics, benralizumab performs a further mechanism beyond the blockage of the IL-5 axis, which is the recruitment of natural killer (NK) cells, macrophages and neutrophils, and the induction of antibody-dependent cell-mediated cytotoxicity (ADCC) for eosinophils and basophils [58]. Despite this, the only single trial performed to investigate the subcutaneous administration of benralizumab 30 mg monthly in patients with EoE over a period of 24 weeks (the MESSINA study; ClinicalTrials.gov Identifier: NCT04543409) did not show a direct correlation between histopathologic and endoscopic improvements assessed through EREFS and clinical amelioration assessed through DSQ [59]. In this regard, it is interesting to note that in a previous study, IL-5R was found to be lower expressed in tissue eosinophils compared to blood eosinophils [60].

In view of the above, the nonlinear correspondence between the histopathologic improvement and the upgrading of the clinical picture may support the hypothesis that the role of eosinophils might be less important than previously thought in sustaining the pathogenesis of EoE, suggesting to focus sharper attention on other inflammatory Th2-like mediators residing in the oesophageal mucosa, such as T-regulatory cells and mast cells.

### 3.4. Tezepelumab: The Anti-Thymic Stromal Lymphopoietin (TSLP) mAb

TSLP is a member of the IL-2 cytokine family and acts as an alarmin, performing a regulation of the Th2 response by driving the production of IL-4, IL-5, IL-9, and IL-13, as well as having pro-inflammatory effects. TSLP is produced by epithelial cells and, at a lower rate, by basophils, mast cells, and dendritic cells of the skin, lung, and gut. Its receptor is a heterodimer consisting of the IL-7 receptor α-chain (IL-7Rα) and the TSLP-R chain [61]. TSLP acts directly on dendritic cells, CD4^+^, and CD8^+^ cells, exerting effects also on granulocytes, mast cells, ILC2s, NK cells, smooth muscle cells, and tumor cells [61,62]. A recent trial showed that in esophageal-derived memory CD4^+^ T cells from patients with EoE, the receptor for TSLP directly responded at a higher rate compared to placebo, as well as circulating memory CD4^+^ T cells. Particularly, TSLP increased the proliferation of CD4^+^ T cells, enhanced type 2 cytokines production, induced the expression of its own receptor, and modulated the expression of several other genes, providing, in this way, a feed-forward loop [63].

Tezepelumab is an IgG2λ human mAb targeting circulating TSLP and preventing its bond to the receptor. It recently received the orphan drug designation from the US FDA for the treatment of EoE [64], and, at the moment, a 52-week trial designed to assess the effectiveness and safety of tezepelumab in adults and adolescents with EoE is ongoing (ClinicalTrials.gov Identifier: NCT05583227).

### 3.5. Lirentelimab: The Anti-Sialic Acid-Binding Immunoglobulin-Like Lectins-8 (SIGLEC-8) mAb

Lirentelimab, also known as AK002, is a humanized non-fucosylated IgG1 antibody against SIGLEC-8, a receptor selectively found on mast cells, eosinophils, and, to a lesser extent, on basophils, that upon activation mediates acute and chronic inflammatory response. Lirentelimab is involved in the inhibition of mast cell activity and in eosinophil reduction mediated by a dual mechanism involving both ADCC and apoptosis [65,66,67]. Moreover, a significant reduction in neutrophils and immune cell-recruiting cytokines, like IL-6, CCL2, CXCL2, IL-13, and TNF, was reported in mice given an anti-SIGLEC-8 mAb and IL-33 [68]. Furthermore, unlike IL-5Rα, the expression of SIGLEC-8 is stable between blood and tissue compartments [66], suggesting it is an important target.

In the ENIGMA trial, patients with eosinophilic gastritis (EoG) and duodenitis (EoD) were randomized 1:1:1 (lirentelimab low dose—up to 1 mg/kg; lirentelimab high dose—up to 3 mg/kg; placebo) and received four monthly infusions. The lirentelimab combined group reported a significant treatment response (reduction >30% in total symptom score and reduction >75% in gastrointestinal eosinophil count). Notably, patients with concomitant EoE and treated with lirentelimab also reported an improvement in dysphagia [69]. Otherwise, the recent KRYPTOS trial evaluated more specifically the effect of lirentelimab high and low dose compared to placebo on patients ≥ 12 years old with EoE, reporting a significant effect of lirentelimab in achieving complete histopathologic remission (88% for high dose, 92% for low dose, and 11% for placebo), even higher when considering only adolescents. Unfortunately, the symptomatic endpoint (mean change in DSQ) did not achieve a proportional improvement [70].

A recent meta-analysis on seventeen randomized control trials comparing the efficacy of all drugs versus each other or placebo in adults and adolescents with active EoE ranked lirentelimab 1 mg/kg monthly as first for histopathologic complete remission, but not for endoscopic and symptomatic remission [71].

### 3.6. Other mAbs

IL-15 is a master immune checkpoint in gut immunology released by several cells of the innate immune system (i.e., dendritic cells and macrophages) but also by fibroblasts and epithelial cells, thus making it another interesting target for the treatment of EoE [72]. IL-15 was found to be particularly expressed in the basal layers of the epithelium in active EoE [73]. CALY-002 is an anti-IL-15 mAb, and it is currently under study to evaluate its safety, tolerability, pharmacokinetics, and pharmacodynamics in patients with EoE or celiac disease (ClinicalTrials.gov Identifier: NCT04593251) [74].

Another ongoing trial (the EvolvE study) is assessing the effect on adults with EoE of barzolvolimab, also known as CDX-0159 [75], a humanized mAb that specifically binds the receptor tyrosine kinase KIT and strongly inhibits its activity, which is required for mast cell function and survival (ClinicalTrials.gov Identifier: NCT05774184) [76].

A previous study aimed to assess the effect of omalizumab, an anti-IgE mAb, reported histological and clinical improvement in only 33% of patients [77], consistent with the concept that EoE is not an IgE-mediated disease; otherwise, it is associated with IgG4 [78]. No significant improvements were reported with infliximab, an anti-TNFα IgG1 mAb [79].

## 4. The Rationale behind the Choice of a Biologic Therapy and Future Perspectives

As already stated, currently, the only biologic therapy approved by the EMA and FDA for the treatment of EoE is dupilumab, usually conceived as a last-line therapy for patients refractory to more traditional ones. Despite the undeniable economic impact of biologic therapies, in the near future, there will be room for further reflection on the opportunity to prescribe this biologic agent as a first-line therapy in selected cases. Particularly, this might prove to be advantageous for patients affected by multiple co-morbid atopic conditions such as asthma, atopic dermatitis, and chronic rhinosinusitis with nasal polyps—CRSwNP, leading both to organic clinical improvement and cushioning the economic inconvenience. From this perspective, it could be interesting also to assess the effects on EoE through real studies involving co-morbid patients treated with biologic agents (i.e., tezepelumab, benralizumab) for other diseases.

Moreover, another feature to consider in the choice of a biologic strategy is the grade of severity of the EoE, which should be built on a multidisciplinary approach combining symptoms, endoscopic findings, and histology. In this regard, the recently developed Index of Severity of Eosinophilic Esophagitis (I-SEE) [80] appears to be a promising tool. The focus of future trials, in fact, should be mandatorily addressed to achieving both histologic and clinically significant improvements. For this reason, the goal of greater homogeneity for the expression of results might make the different biologic agents more comparable with each other and with other therapies.

## 5. Conclusions

**Figure 1:**
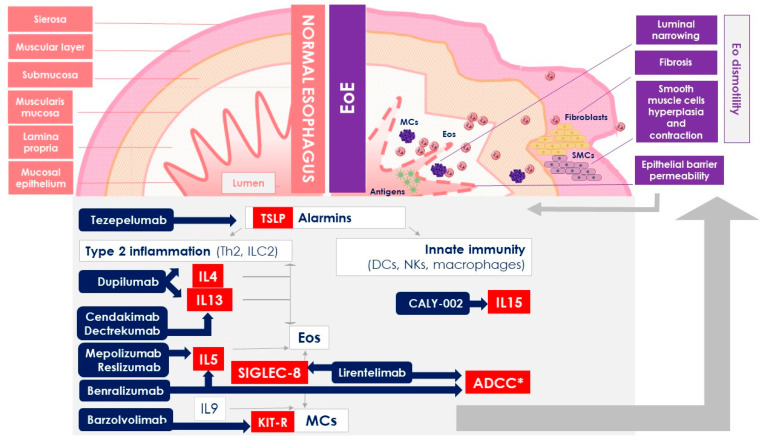
Histopathologic alterations and different pathways targeted by biologic drugs investigated for the treatment of EoE. Abbreviations: ADCC—antibody-dependent cell-mediated cytotoxicity; EoE—eosinophilic esophagitis; Eos—eosinophils; KIT-R—KIT-receptor; MCs—mast cells; NKs—natural killer cells; SMCs—smooth muscle cells; TSLP—thymic stromal lymphopoietin. * ADCC is not a specific molecular target but rather a mechanism involved in the lowering of eosinophil levels.

The effective approach to EoE requires the consideration of several inflammatory mechanisms involved in the pathogenesis. From this perspective, biologic drugs targeting these different pathways (Figure 1) may represent an important option in the treatment of EoE. Recent findings about the ability of some mAbs (first of all, dupilumab) to counter this disease are very encouraging (Table 1). Nevertheless, the road to permanent success is still long, and for this reason, further studies will be needed to determine the long-term effects of these mAbs and to discover new potential targets.

## Figures and Tables

**Table 1:** Monoclonal antibodies investigated for the treatment of EoE. NS = non significant.

mAb	Target	Completed Trials (No. of Patients)	Histologic Complete Remission (≤6 eos/hpf)	Endoscopic Improvement	Clinical Outcome	Ongoing Trials
Dupilumab	IL-4Rα (IL-4, IL-13)	Dellon et al. [33] (321)	Yes	Yes (EREFS)	Yes (DSQ)	NCT04394351
		Dellon et al. [35] (46)	Yes	Yes (EREFS; pre-dilation esophageal diameter)	Yes	NCT06101095 NCT05247866
Cendakimab (aka RPC4046 or CC-93538)	IL-13	Hirano et al. [41] (99) Dellon et al. (extension) [42] (66)	Yes (EoEHSS)	Yes (EREFS)	Partial	NCT04753697 NCT06101095 NCT05175352
Dectrekumab (aka QAX576)	IL-13	Rothenberg et al. [48] (25)	NS	-	No	
Mepolizumab	IL-5	Stein et al. [52] (4)	NS	Yes in 3/4	Yes (dysphagia, QoL scores)	
		Straumann et al. [53] (11)	NS	NS	NS	
		Assa’ad et al. [54] (59)	NS	Yes	NS	
		Dellon et al. [55] (66)	Yes	Yes (EREFS)	NS (EEsAI)	
Reslizumab	IL-5	Spergel et al. [56] (226)	NS	-	NS	
		Markowitz et al. [57] (12)	Yes	No progression	Yes	
Benralizumab	IL-5-R	NCT04543409 [59] (211)	Yes	Yes (EREFS)	NS (DSQ)	
Tezepelumab	TSLP	-	-	-	-	NCT05583227
Lirentelimab	SIGLEC-8	Dellon et al. [70] (276)	Yes	-	NS (DSQ)	
CALY-002	IL-15	-	-	-	-	NCT04593251
Barzolvolimab	KIT	-	-	-	-	NCT05774184
Omalizumab	IgE	Loizou et al. [77] (17)	NS	NS	NS	
Infliximab	TNF	Straumann et al. [79] (3)	NS	NS	NS	

## Data Availability

No new data were created.
